# Association between infection prevention and control safety culture and healthcare workers’ compliance with infection control measures: a cross-sectional study

**DOI:** 10.3389/fpubh.2025.1668493

**Published:** 2025-10-17

**Authors:** Yisui Cen, Chuyu Lao, Zihuan Li, Huiwen Zhao, Tian Wang, Cuiqiong Fan, Baohong Liu, Zhenyao Zhao, Ya Zou, Guanwen Lin

**Affiliations:** ^1^Department of Infection Prevention and Control, The Affiliated Guangdong Second Provincial General Hospital of Jinan University, Guangzhou, China; ^2^Department of Pharmacy, The First Affiliated Hospital of Sun Yat-sen University, Guangzhou, China; ^3^, Shenzhen People's Hospital, The First Affiliated Hospital, Southern University of Science and Technology, The Second Clinical Medical College, Jinan University, Shenzhen, China; ^4^Faculty of Humanities and Social Sciences, Hong Kong Metropolitan University, Hong Kong, China

**Keywords:** infection control, patient safety, healthcare worker, surveys and questionnaires, hospital safety climate

## Abstract

**Background:**

Infection prevention and control (IPC) safety culture is recognized as a crucial factor in improving healthcare workers’ (HCWs) compliance with IPC measures. Despite its importance, evidence regarding the influence of IPC safety culture on compliance remains limited and warrants further investigation.

**Methods:**

A cross-sectional study was conducted from March 27 to April 3, 2025, using an online questionnaire to examine the association between IPC safety culture and HCWs’ compliance with IPC measures across more than 200 healthcare institutions, including 12 tertiary hospitals, in Haizhu District, Guangzhou, Guangdong Province, China. IPC safety culture was assessed using the Infection Control Culture Scale, while compliance with IPC measures was measured with the Compliance with Standard Precautions Scale, both utilizing five-point Likert scales. Student’s t test assessed differences between groups, and linear correlation analyses examined relationships between IPC safety culture dimensions and compliance scores. Multivariate logistic regression was used to identify factors associated with HCWs’ compliance to IP measures.

**Results:**

A total of 1,600 questionnaires were distributed, of which 1,471 were valid (91.94%). HCWs aged over 30 years, with more than 5 years of work experience, and holding a Master’s degree or higher had higher IPC safety culture scores compared with their counterparts (*p* < 0.05). Similarly, HCWs aged over 30 years, with more than 5 years of experience, a Master’s degree or higher, and possessing clinical teaching qualifications demonstrated higher compliance with IPC measures compared with their counterparts (*p* < 0.05). Linear correlation analysis showed that basic IPC competence was strongly correlated with all dimensions of IPC compliance (*r* = 0.70, 0.63, 0.62, 0.63, 0.64, and 0.58, *p* < 0.001). Hospital management climate, departmental team cooperation, organizational learning and continuous improvement, and reporting frequency of hospital-acquired infections (HAIs) adverse events were weakly to moderately correlated with IPC compliance (*p* < 0.001). Based on a median score of 4.95 (IQR 4.77–5.00), 840 participants (57.10%) were classified as having good compliance, and 631 (42.90%) as poor compliance. Multivariate logistic regression indicated that clinical instructor status (OR = 1.60, 95% CI: 1.46–1.79), basic IPC competence (OR = 1.87, 95% CI: 1.73–1.99), hospital management climate (OR = 1.50, 95% CI: 1.08–2.10), reporting frequency of HAIs adverse events (OR = 1.36, 95% CI: 1.19–1.54), and organizational learning and continuous improvement (OR = 3.59, 95% CI: 2.68–4.80) as independent predictors of IPC compliance.

**Conclusion:**

IPC safety culture significantly affects HCWs’ adherence to IPC measures. Enhancing basic IPC competence, hospital management support, organizational learning, reporting practices, and targeted interventions for clinical instructors can improve compliance and help prevent HAIs.

## Introduction

Infection prevention and control (IPC) is fundamental to patient safety and healthcare quality. Effective IPC requires healthcare workers (HCWs) to consistently adhere to standardized practices, including hand hygiene, use of personal protective equipment, sharps injury prevention, environmental cleaning, and medical waste management (1–4). Compliance with these measures is strongly influenced by organizational culture factors such as leadership, team cooperation, and communication, which collectively shape HCWs’ adherence and overall IPC performance (5–8).

Several instruments have been developed to measure HAI safety culture. Pogorzelska-Maziarz et al. (32) adapted the Leading a Culture of Quality (LCQ) scale to create the LCQ in Infection Prevention (LCQ-IP). Studies using LCQ-IP reported moderate safety culture scores (3.37–3.86), with factors such as clinical experience, nationality, and training influencing results (9–11). However, LCQ-IP lacks a theoretical framework, and its dimensions and items are insufficiently justified, limiting generalizability. To address these limitations, Chinese researchers adapted the Hospital Survey on Patient Safety Culture (HSOPSC) through expert consultation to develop the Infection Control Culture Scale (ICCS), though its dimension construction also lacks a clear theoretical basis.

Most existing studies focus on a few core elements of HAI safety culture rather than examining all dimensions and their impact on HCWs’ compliance with IPC measures. The mechanisms through which IPC safety culture influences adherence remain unclear, representing a significant research gap. Systematic investigation is needed to clarify how different dimensions of infection control culture affect HCWs’ compliance, providing evidence for targeted interventions to improve patient safety.

## Materials and methods

### Participants

This cross-sectional study was conducted using an online survey to assess IPC safety culture and compliance with IPC measures among HCWs in over 200 healthcare institutions in Haizhu District, Guangzhou, Guangdong Province, China, including 12 tertiary hospitals. Participants were enrolled through a retrospective questionnaire-based survey conducted from March 27 to April 3, 2025. HCWs were invited to participate via an online questionnaire. Participation was entirely voluntary, and no compensation or incentives were provided. Because the survey relied on voluntary participation, the number of participants from each institution varied, although each institution was required to contribute at least two participants. Individuals whose primary roles were limited to non-clinical hospital operations, such as finance, security, medical records management, or library services were excluded from the study. Collected socio-demographic data included sex, age, occupation type, years of work experience, professional title, education level, hospital affiliation, possess clinical teaching qualifications, and average weekly working hours.

### Ethics approval and consent statement

The study was approved by the Ethics Committee of The Affiliated Guangdong Second Provincial General Hospital of Jinan University, Guangzhou, China (Approval no. 2025-KY-KZ-257-01). As this study utilized a de-identified database, the Ethics Committee of the Affiliated Guangdong Second Provincial General Hospital of Jinan University waived the requirement for informed consent. The purpose of the study was clearly explained on the opening page of the online questionnaire, and only participants who provided informed consent were able to proceed with the survey. All procedures in this study were conducted in accordance with relevant ethical guidelines and regulations.

### The scale of infection control culture assessment

Dandan Zhang and her colleagues from Huazhong University of Science and Technology adapted the Hospital Survey on Patient Safety Culture (12, 13), originally developed by researchers at Westat under a contract with the Agency for Healthcare Research and Quality, through expert consultation to create the Infection Control Culture Scale (ICCS). The ICCS consists of seven dimensions: basic IPC competence, hospital management climate, departmental team cooperation, reporting frequency of hospital-acquired infection adverse events, leadership attention, organizational learning and continuous improvement, and workload. These seven dimensions comprise a total of 30 indicators (Supplementary Table S1), each rated on a five-point Likert scale ranging from “strongly disagree” to “strongly agree.” The overall Cronbach’s *α* coefficient of the scale is 0.980, and the split-half reliability coefficient is 0.893. The Cronbach’s α for each dimension exceeds 0.8, indicating high internal consistency and reliability of the instrument. In this study, we obtained authorization from all original authors to use the ICCS scale and conducted the research in accordance with the relevant guidelines and requirements.

### Compliance with standard precautions scale

The Compliance with Standard Precautions Scale (14, 15) encompasses the core elements of compliance with IPC measures, including the use of personal protective equipment, prevention of sharps injuries, proper medical waste disposal, environmental disinfection, and prevention of cross-contamination. This scale has demonstrated strong reliability and validity and is widely used in compliance with IPC measures. It utilizes a five-point Likert scale to assess varying levels of compliance, with response options ranging from “never” “occasionally” “about half of the time” “most of the time” to “always.”

### Statistical analyses

All statistical analyses were performed using IBM SPSS statistical software (version 29.0). Counted data were described by the number of cases (percentage), and the Kolmogorov–Smirnov test was used to verify the normality of the data. For continuous variables that conformed to a normal distribution, the mean and 95% confidence intervals were visualized using GraphPad Prism (version 9.5.0), and differences between groups were evaluated using Student’s *t-*test. Simple linear correlation analysis was performed to calculate the correlation coefficient (r). Variables with a *p* < 0.05 in the univariate (bivariate) analysis were included as candidate variables for the multivariate logistic regression. Following the transformation of IPC compliance scores into a binary variable, a logistic regression model was used to examine the association between IPC safety culture and compliance with IPC measures. Compliance was dichotomized based on the median score: participants with scores below the median were classified as having poor compliance, while those with scores above the median were considered to have good compliance. All statistical analyses were evaluated at the statistical significance level of *p* < 0.05 (two-sided).

## Results

### Demographic and professional characteristics of the study population

A total of 1,600 questionnaires were distributed. After excluding invalid responses, 1,471 valid questionnaires (91.94%) were collected, representing the final sample size of HCWs. The majority were female (80.42%) and aged over 30 years (59.42%). Most participants were employed at primary care hospitals (78.25%) and worked primarily as nurses (62.27%). Respondents with <5 years of work experience accounted for 52.96%. In terms of professional rank, 86.06% held intermediate titles or below, and 93.07% had an educational background of a bachelor’s degree or below. Additionally, 70.56% of participants did not possess clinical teaching qualifications. More than half (55.88%) reported working over 40 h per week. The detailed demographic characteristics are presented in Table 1.

**Table 1:** The essential demographic data of the study population.

Characteristic	Number (*n* = 1,471)	%
Sex		
Man	288	19.58
Female	1,183	80.42
Age, year		
≤ 30	597	40.58
> 30	874	59.42
Hospital		
Tertiary hospital	320	21.75
Primary care hospital	1,151	78.25
Job position		
Doctor	555	37.73
Nurse	916	62.27
Years of work experience		
≤ 5	779	52.96
> 5	692	47.04
Professional titles		
Intermediate level and below	1,266	86.06
Associate senior level and above	205	13.94
Educational background		
Bachelor’s degree and below	1,369	93.07
Master’s degree and above	102	6.93
Possess clinical teaching qualifications		
Yes	433	29.44
No	1,038	70.56
Weekly working hours		
≤ 40 h	649	44.12
> 40 h	822	55.88

### Scores of IPC safety culture and compliance with IPC measures

**Figure 1:**
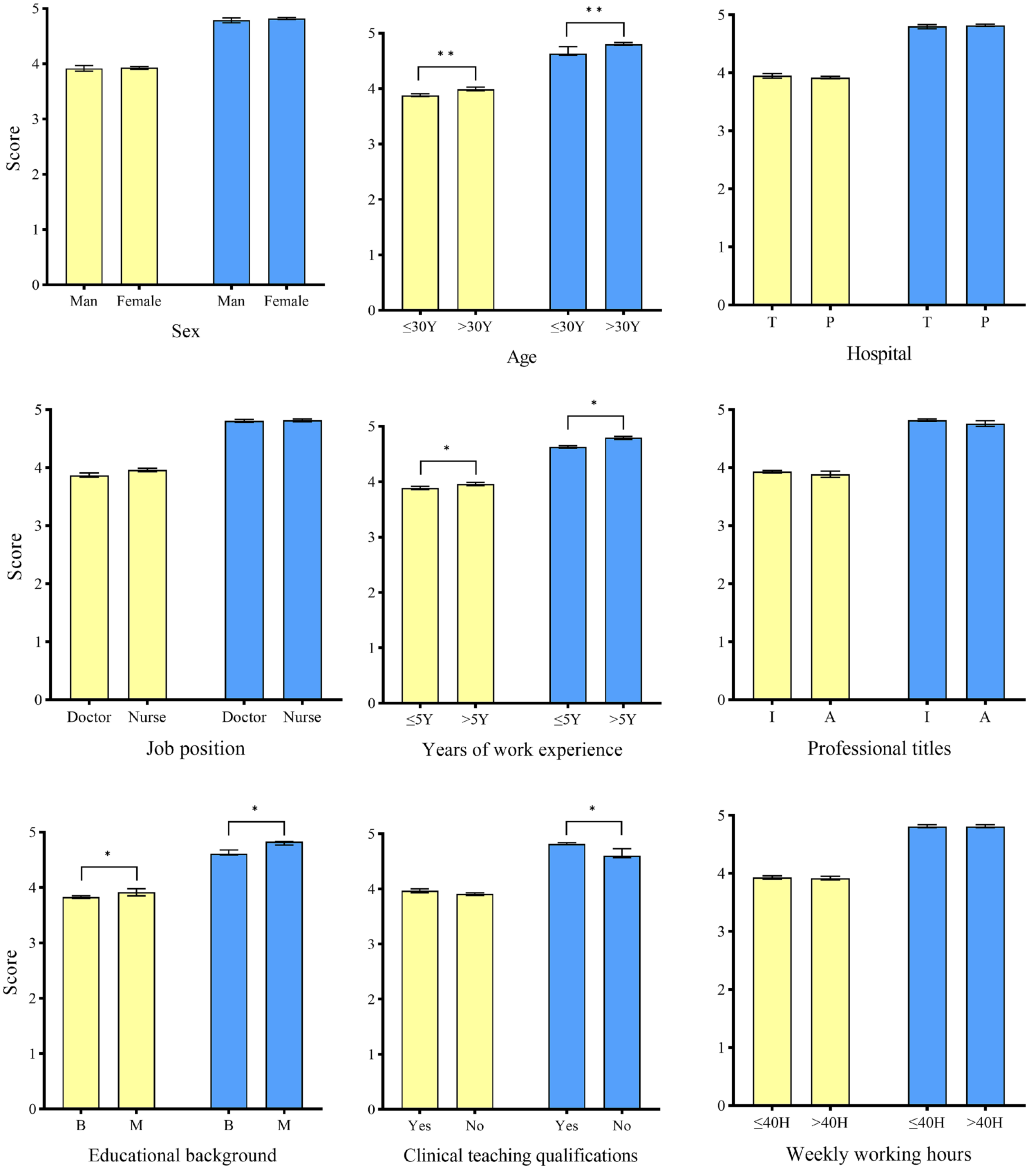
Scores of IPC safety culture and compliance with IPC measures among healthcare workers with different characteristics. The yellow color represents IPC safety culture. The blue color represents compliance with IPC measures. T: Tertiary hospital. P: Primary care hospital. I: Intermediate level and below. A: Associate senior level and above. B: bachelor’s degree and below. M: master’s degree and above. Student’s t test was used to evaluate the differences between each item. Symbols for *p*-values: * < 0.05, ** < 0.001.

Statistically significant differences in IPC safety culture scores were observed among HCWs across different age groups, years of work experience, and educational backgrounds. Likewise, compliance with IPC measures scores varied significantly by age group, years of work experience, educational background, and clinical teaching qualifications (Figure 1).

### The linear correlation analysis results between the scores of each dimension of the IPC safety culture and measures compliance

In the correlation analysis between IPC safety culture and compliance with IPC measures, Basic IPC competence demonstrated a strong linear relationship with all dimensions of compliance with IPC measures (*r* = 0.70, 0.63, 0.62, 0.63, 0.64, and 0.58, *p* < 0.001). Hospital management climate, departmental team cooperation, organizational learning and continuous improvement, and reporting frequency of hospital-acquired infection adverse events showed weak to moderate correlations with IPC compliance (*p* < 0.001). In contrast, leadership attention and workload were only weakly correlated with IPC compliance (Table 2).

**Table 2:** The linear correlation analysis results between the scores of each dimension of the IPC safety culture and measures compliance.

IPC safety culture	Compliance with IPC measures					
	B1	B2	B3	B4	B5	B6
A1	0.70^b^	0.63^b^	0.62^b^	0.63^b^	0.64^b^	0.58^b^
A2	0.43^b^	0.42^b^	0.46^b^	0.39^b^	0.40^b^	0.36^b^
A3	0.33^b^	0.31^b^	0.31^b^	0.27^b^	0.29^b^	0.25^b^
A4	0.36^b^	0.36^b^	0.38^b^	0.33^b^	0.37^b^	0.32^b^
A5	0.19^a^	0.19^a^	0.26^b^	0.27^a^	0.17^a^	0.18^a^
A6	0.50^b^	0.42^b^	0.44^b^	0.38^b^	0.42^b^	0.36^b^
A7	0.07^a^	0.07^a^	0.11^b^	0.07^a^	0.06^a^	0.04^a^

A1: Basic IPC competence. A2: Hospital management climate. A3: Departmental team cooperation. A4: Reporting frequency of hospital-acquired infection adverse events. A5: Leadership attention. A6: Organizational learning and continuous improvement. A7: Workload. B1: Hand hygiene. B2: Wearing gloves. B3: Masks, face shields, and isolation gowns. B4: Needlestick and sharps injuries. B5: Environmental cleaning and disinfection. B6: Disposal of medical waste. Symbols for ^a^*p* < 0.05, ^b^*p* < 0.001.

### Multivariate analysis of IPC safety culture and compliance with IPC measures

The median score for compliance with IPC measures was 4.95 (IQR 4.77–5.00). A total of 840 participants (57.10%) with scores ≥ 4.95 were defined as having good compliance, while 631 participants (42.90%) with scores < 4.95 were categorized as having poor compliance. Variables with statistical significance in the univariate analysis were included in the logistic regression model. Results showed that clinical instructors demonstrated better compliance with IPC measures (OR = 1.60, 95% CI: 1.46–1.79). Higher compliance was also associated with basic IPC competence (OR = 1.87, 95% CI: 1.73–1.99), hospital management climate (OR = 1.50, 95% CI: 1.08–2.10), reporting frequency of hospital-acquired infection adverse events (OR = 1.36, 95% CI: 1.19–1.54), and organizational learning and continuous improvement (OR = 3.59, 95% CI: 2.68–4.80), as shown in Table 3.

**Table 3:** Multivariate analysis of IPC safety culture and compliance with IPC measures.

Variable	*β*	Wald	*P*	OR	OR 95%CI
Clinical instructor	0.51	13.30	< 0.001	1.60	1.46–1.79
IPC safety culture					
A1	0.44	5.06	0.011	1.87	1.73–1.99
A2	0.41	5.72	0.017	1.50	1.08–2.10
A4	0.30	21.54	< 0.001	1.36	1.19–1.54
A6	1.28	73.34	< 0.001	3.59	2.68–4.80

Clinical instructor status was included in the model, with “No” as the reference category. A1: Basic IPC competence. A2: Hospital management climate. A4: Reporting frequency of hospital-acquired infection adverse events. A6: Organizational learning and continuous improvement. OR, Odds Ratio; CI, Confidence Interval.

## Discussion

This study is the first to examine the association between IPC safety culture and HCWs’ compliance with IPC measures in healthcare institutions in Haizhu District, Guangzhou, Guangdong Province, China. The findings indicate that key components of IPC safety culture, including basic IPC competence, hospital management climate, frequency of reporting hospital-acquired infection adverse events, and organizational learning and continuous improvement, are significantly associated with HCWs’ adherence to IPC practices.

The establishment of surveillance systems is an important component of IPC (16–18), however, HCWs’ individual behaviors and personal initiative also play a decisive role in infection prevention and control (19–23). In this study, age, educational background, years of work experience, and clinical teaching qualifications were associated with higher compliance scores in IPC. With increasing years of work experience, HCWs were more likely to have received targeted IPC training and assessments. Moreover, those with longer service and clinical teaching qualifications experience exhibited more positive attitudes toward IPC, suggesting that greater clinical experience and increased responsibilities foster a stronger sense of accountability and attention to infection control. This may be attributed to a better understanding of the long-term benefits of IPC practices and increased confidence in the effectiveness of these measures (24, 25). Additionally, the findings also indicated that HCWs with higher educational attainment scored better in both IPC safety culture and compliance with IPC measures. Therefore, improving the overall educational level of HCWs or offering more targeted training opportunities for those with below educational backgrounds may be effective strategies to enhance infection control practices across healthcare settings (26).

The dimension of organizational learning and continuous improvement includes four key components: departmental reviews of HAIs adverse events, monthly IPC training sessions conducted by infection control observers, open reporting of potential infection risks by staff, and timely feedback following the reporting of HAIs incidents. However, most clinical departments lack systematic and professional training in the management of HAIs events. In contrast, infection control observers (27) act as effective coaches or facilitators, providing guidance throughout the change process, addressing resistance, and remaining aware of how their actions impact the individuals and teams they work with (28, 29). Their ability to deliver timely feedback and conduct regular training highlights that ongoing, structured education and continuous improvement initiatives are essential for enhancing the competencies of frontline HCWs (30). Additionally, the integration of simulation-based training with hands-on clinical education has been shown to significantly enhance compliance with infection control protocols (31). Consequently, fostering organizational learning and continuous improvement is essential for strengthening adherence to IPC measures.

### Limitations

This study has several limitations. First, its cross-sectional design limits the ability to establish causal relationships between IPC safety culture and HCWs’ compliance with IPC measures. Future longitudinal or interventional studies are needed to further explore causal effects. Second, convenience sampling is often employed in qualitative research to facilitate data collection. However, it does not ensure sample adequacy and may introduce selection bias. Thrid, self-reported data may be subject to recall or social desirability bias, which could influence the accuracy of the results. Finally, HCWs’ compliance to IPC measures was assessed using a quantitative self-administered questionnaire. While this method allows efficient collection of data from a large number of participants, it may not fully capture actual IPC practices in the clinical setting. Actual practice of IPC among HCWs cannot be comprehensively assessed through a self-administered questionnaire, as this approach may lead to systematic bias. Observational or qualitative approaches might more accurately reflect HCWs’ real compliance to IPC measures during routine patient care.

## Conclusion

Our findings indicate that IPC safety culture significantly influences HCWs’ adherence to IPC measures. Strengthening Basic IPC competence, hospital management support, organizational learning, and reporting practices, along with targeted interventions for clinical instructors, can effectively improve compliance and contribute to the prevention of healthcare-associated infections.

## Data Availability

The original contributions presented in the study are included in the article/Supplementary material, further inquiries can be directed to the corresponding author.
