# Supplementary material for: Association between infection prevention and control safety culture and healthcare workers’ compliance with infection control measures: a cross-sectional study
Source: Front Public Health. 2025 Oct 17;13:1668493. doi: 10.3389/fpubh.2025.1668493 (PMC12575312; doi:10.3389/fpubh.2025.1668493)
Supplement: Supplementary file 1 [file Table_1.doc]

**Supplementary Material**

**Supplementary Table S1 The scale of infection control culture assessment**

| **Dimension 1** | **Basic IPC competence** |
| --- | --- |
| Indicator 1 | I am very clear about the steps and timing of hand hygiene. |
| Indicator 2 | I know how to manage it when a needlestick injury occurs. |
| Indicator 3 | I am familiar with the reporting procedure for infection cases. |
| Indicator 4 | I am aware of the specific isolation measures that should be implemented for patients with multidrug-resistant infections. |
| **Dimension 2** | **Hospital management climate** |
| Indicator 5 | The hospital management provides a work environment that promotes infection control safety. |
| Indicator 6 | The hospital periodically conducts continuing education training on hospital infection prevention. |
| Indicator 7 | The hospital treats infection outbreaks as a veto criterion when selecting advanced departments. |
| Indicator 8 | The department discusses methods to prevent the recurrence of infection-related adverse events. |
| Indicator 9 | The hospital management can provide the necessary infection control supplies for the departments. |
| Indicator 10 | The management only pays attention after an infection-related adverse event occurs. |
| Indicator 11 | When patients with multidrug-resistant infections are transferred between departments, handover problems may occur. |
| **Dimension 3** | **Departmental team cooperation** |
| Indicator 12 | We are actively taking measures to prevent hospital-acquired infections. |
| Indicator 13 | After an infection occurs, we conduct case discussions. |
| Indicator 14 | Infection control physicians and infection control nurses collaborate to carry out infection control work. |
| Indicator 15 | The fact that infection outbreaks have not occurred is entirely due to luck. |
| **Dimension 4** | **Reporting frequency of hospital-acquired infection adverse events** |
| Indicator 16 | Reporting frequency of adverse events after they occur. |
| Indicator 17 | Reporting frequency of potential adverse events that may affect patients. |
| Indicator 18 | Reporting frequency of potential adverse events that may affect staff. |
| **Dimension 5** | **Leadership attention** |
| Indicator 19 | Worried that infection-related adverse events will be recorded in personal files. |
| Indicator 20 | Leadership seriously considers staff suggestions on hospital infection prevention and control. |
| Indicator 21 | Leadership summarizes hospital-acquired infection adverse events that occur in the department. |
| Indicator 22 | Leadership ignores repeatedly occurring hospital-acquired infection adverse events. |
| Indicator 23 | If no hospital-acquired infection adverse events occur, my supervisor will praise me. |
| **Dimension 6** | **Organizational learning and continuous improvement** |
| Indicator 24 | The department summarizes hospital-acquired infection adverse events that occur. |
| Indicator 25 | The infection control officer in the department holds monthly hospital infection training. |
| Indicator 26 | Staff will report any potential hospital-acquired infection risks they discover. |
| Indicator 27 | After reporting hospital-acquired infection adverse events, we receive feedback. |
| **Dimension 7** | **Workload** |
| Indicator 28 | We have enough staff to carry out infection control work. |
| Indicator 29 | Long working hours prevent us from providing the best care. |
| Indicator 30 | Being in an "infection control crisis mode," I always feel that our efforts are insufficient. |
